# An Arthropod Hormone, Ecdysterone, Inhibits the Growth of Breast Cancer Cells via Different Mechanisms

**DOI:** 10.3389/fphar.2020.561537

**Published:** 2020-10-30

**Authors:** O. Shuvalov, O. Fedorova, E. Tananykina, Y. Gnennaya, A. Daks, A. Petukhov, N. A. Barlev

**Affiliations:** ^1^ Institute of cytology, Russian Academy of Sciences (RAS), St-Petersburg, Russia; ^2^ Almazov National Medical Research Centre, St-Petersburg, Russia; ^3^ Moscow Institute of Physics and Technology, Dolgoprudny, Russia; ^4^ Orekhovich Institute of Biochemical Medicine, Moscow, Russia

**Keywords:** ecdysterone, breast cancer, doxorubicin, autophagy, energy metabolism, synergism, doxorubicin, triple negative breast cancer, combination index, dose reduction index, 2-deoxyglucose, extracellular acidification rate, oxygen consumption rate, multiple drug resistance

## Abstract

Ecdysterone (Ecdy) is a hormone found in arthropods, which regulates their development. It is also synthesized by a number of plants to combat insect pests. It provides a number of beneficial pharmacological effects including the anabolic and adaptogenic ones. Ecdysterone is widely marketed as food supplement to enhance the physical performance of athletes. In addition to the estrogen receptor beta (ERbeta)-dependent anabolic effect of Ecdy in muscles, the molecular mechanisms of the plethora of other Ecdy-induced pharmacological effects remain unknown. The aim of this study was to investigate the pharmacological effect of ecdysterone on human breast cancer cell lines of different molecular subtypes. Surprisingly, in contrast to the anabolic effect on muscle tissues, we have revealed a tumor suppressive effect of Ecdy on a panel of breast cancer cell lines studied. Using the SeaHorse-based energy profiling, we have demonstrated that Ecdy dampened glycolysis and respiration, as well as greatly reduced the metabolic potential of triple negative breast cancer cell lines. Furthermore, we have revealed that Ecdy strongly induced autophagy. As part of the combined treatment, based on the Combination Index (CI) and Dose Reduction Index (DRI), Ecdy synergized with doxorubicin to induce cell death in several breast cancer cell lines. In contrast, Ecdy had only minor effect on non-transformed human fibroblasts. Collectively, our results indicate that ecdysterone can be considered as a new potential adjuvant for genotoxic therapy in treatment of breast cancer patients.

## Introduction

Ecdysteroids constitute a class of steroid hormones found in arthropods, which regulate their development including molting and reproduction. Co-evolution of plants and its pathogens and animals, including insects, has generated a plethora of different biochemical pathways allowing plants to synthesize various protective compounds that defend them from various environmental insults.

Thus, Rausher (2001) about 6% of plant species (ferns, angiosperms and gymnosperms) synthesize ecdysteroids (phytoecdysteroids) as protective mechanisms against insects (Dinan, 2001). To date, 517 different ecdysteroids derived from both plants and insects have been described and listed in the ecdysteroid database (EcdyBase, www.ecdybase.org).

A number of studies have shown that ecdysteroids partake in different biological activities within humans (Lafont and Dinan, 2003; Dinan et al., 2009; Isenmann et al., 2019). However, the pharmacological potential of the vast majority of ecdysteroids remains to be elucidated. The most studied one is 20-Hydroxyecdysone or Ecdysterone (Ecdy). Extracts of Ecdy produced by *Leuzea carthamoides* are widely marketed as various diet supplements for athletes.

Early pharmacological experiments have shown that it has a low toxicity in mammals (LD 50 > 9 g/kg) (Ogawa et al., 1974; Lafont and Dinan, 2003). The maximum recommended dose of Ecdy for athletes is 500–1,000 mg per day (Dinan and Lafont, 2006). The positive pharmacological effects of ecdysterone on humans are well documented and include: anabolic, anti-diabetic, neuron protective, anti-angiocardiopathological, immune-stimulating, antidepressant to name a few (for a comprehensive review, see (Lafont and Dinan, 2003; Dinan et al., 2009; Bajguz et al., 2015).

Ecdysterone attracts the most attention as a natural anabolic and adaptogenic compound. It is widely marketed as a “natural anabolic agent” to athletes, in the form of dietary supplements which increase strength and muscle mass during resistance training, to reduce fatigue, and to ease recovery (Isenmann et al., 2019). A number of papers have shown an ecdysterone-mediated increase in sport performance among both mice and humans (Azizov and Seĭfulla, 1998; Gorelick-Feldman et al., 2008; Parr et al., 2015; Isenmann et al., 2019). These studies demonstrated the anabolic effect of ecdysterone and its benefical effects to athletes, and contributed to ecdysterone being considered as a potential enhancement substance in anti-doping control (Parr et al., 2020). Since December 2019, ecdysterone is in the focus of WADA (World Anti-doping Agency) investigations.

In insects, ecdysterone acts in nanomolar concentrations through ecdysone nuclear receptors (EcR). However, this compound does not display any hormonal activity in humans because they have no EcRs, nor it interacts with androgen or glucocorticoid receptors. However, ecdysterone was shown *in vitro* to stimulate ERbeta, which is involved in skeletal muscle hypertrophy (Parr et al., 2014).

Considering the diversity of ecdysterone-mediated pharmacological activities in the human organism, including different tissues (see Dinan and Lafont, 2006), it seems that the ERbeta-signaling pathway is not the only molecular mechanism which is utilized by ecdysterone. However, other molecular mechanisms underlying the broad spectrum of ecdysterone-mediated pharmacological effects remain elusive.

Unlike for other hormone-like compounds, very little information is available about the effects of ecdysterone on cancer cells and malignancies. It is interesting to note that despite the reported anabolic properties of ecdysterone regarding sport performance, several studies described ecdysterone-induced sensitization of cancer cells to genotoxic drugs and reduction of tumors in mice (Konovalova et al., 2002; Martins et al., 2015).

Here, we provide evidence that ecdysterone can inhibit the proliferation of breast cancer cells. Mechanistically, it down-regulates the metabolic potential of cancer cells and induces autophagy. Moreover, ecdysterone strongly sensitizes breast cancer cells to doxorubicin, resulting in a significant reduction of the effective dose of doxorubicin. Importantly, the effect of ecdysterone on non-transformed human fibroblasts was minimal.

## Materials and Methods

### Сell Lines and Reagents

All the cell lines used in this study (MCF7, MDA-MB-231, MDA-MB-468, DF2 and WI-38) were purchased from ATCC. Cells were grown in DMEM media supplemented with 10% fetal bovine serum, 100 μg/ml gentamycin, and 2 mM l-glutamine. To grow MCF7 cells the medium was also supplemented with 10 μg/ml insulin (NM Penfild, Denmark). Cells were grown at 37°C in 5% CO_2_ atmosphere.

Ecdysterone (95% purity, Frog Tech, Russia) was dissolved in DMSO. Thus, DMSO was used as a control for all experiments with ecdysterone (0 μM Ecdy). Doxorubicin (98% purity, Sigma, United States) and 2-DG (98% purity, Sigma, United States) were dissolved in water.

### MTT Assay

For MTT experiments, 10,000 cells were planted overnight in each well of a 96-well plate. 10 wells per sample were used. A day after, ecdysterone or (and) doxorubicin were added in the required concentrations for 48 h. For cells treated with ecdysterone, DMSO was used as a control. Then 10 μL of 5 mg/ml Thiazolyl Blue (Paneko, Russia) solution was added to each well and cells were kept for 3,5 h at 37°С in CO_2_ incubator. After removing the thiazol-containing medium, 150 μL isopropyl alcohol (supplemented with 40 mM HCl and 0,1% NP-40) was added to dissolve the MTT-formazan salt. The absorbance at 570 and 630 nm (reference) was measured using BioRad iMark microplate reader (BioRad, United States). Results are represented as the mean ± SD.

### Colony-formation Assay

To perform colony-formation assay, 1,000 cells were planted per well on a 6-well plate, in triplicates. 24 h later, the cells were treated with 0, 100, 150, 250, 350 or 500 μM ecdysterone for 96 h. After treatment, fresh media was added, and cells were grown for 10 days. After the indicated time, cells were fixed with acetic acid/methanol (1:7, v/v) and stained with 0.5% crystal violet. The number of colonies was calculated. Results are represented as the mean ± SD of three biological replicates.

### Proliferation Assay

About 25,000 cells were seeded in 12-well plates and incubated with different amounts of 0–750 µM Ecdy for 4 days. Following the incubation, cells were trypsinized, stained with trypan blue and calculated using hemocytometer. Six replicates were used for analysis. Results are calculated as the mean ± SD; *p < 0.05.

### Cell Cycle Analysis

Flow cytometry analysis of cell cycle distribution was done essentially as described previously (Lezina et al., 2014). A day after seeding, cells were treated with ecdysterone (0, 250, 500, or 750 μM) for 48 h in triplicates. After harvesting, cells were washed once with PBS, and fixed in 70% ethanol at −20°C for 1 h. The 30 min staining of DNA content was carried out by using 50 μg/ml of PI (Invitrogen, United States) and 1 μg/ml RNase A (ThermoFischer). Samples were analyzed by CytoFLEX (Beckman Coulter, United States) flow cytometer. Results were processed by CyteExpert software (Beckman Coulter, United States).

### SeaHorse Energy Profiling

To perform the energy profiling using SeaHorse apparatus, 30,000 cells were seeded to each well (except for the background wells) of a 24-well SeaHorse plate (Agilent, United States) overnight. Four wells were used per sample. Then, 0–1,000 μM Ecdy was added for 48 h. 12 h before analysis, the Sensor Cartridge was equilibrated in XF Calibrant (Agilent, United States) at 37°C in a non-CO_2_ incubator. SeaHorse XF Energy Phenotype kit (Agilent, United States) was applied for assay. SeaHorse XF base medium was supplemented with 1 mM pyruvate, 2 mM glutamine and 10 mM glucose, pH 7.4. Stressor mix consisting of olygomycin and FCCP (both Agilent, United States) was used to achieve final concentrations 1 and 2 μM in wells, respectively. Assay was run in the XFe24 Analyser device (Agilent, United States) in accordance with the manufacturer’s instructions. Data were normalized using total protein quantification by BCA assay (ThermoFischer, United States) and processed by SeaHorse XF Cell Energy Phenotype Test Report Generator (Agilent, United States). Results are represented as the mean ± SEM.

### Analysis of Apoptosis and Total Cell Death

Flow cytometric determination of cell death including apoptosis was carried out by using annexin V-FITC/(PI or 7-AAD) double staining. To analyze the influence of ecdysterone on cell death, annexin V–FITC/PI kit (BD Biosciences, United States) was used, whereas in studies of combined treatments (doxorubicin and ecdysterone) annexin V–FITC/7AAD (ThermoFischer, United States) was applied in accordance with the corresponding manufacturer’s protocols. Cells were treated for 48 h with ecdysterone (0, 250, 500, and 750 μM) and doxorubicin (0, 0.15, and 0.25 μM) separately or in combination. A minimum of 5,000 cells were analyzed by CytoFLEX (Beckman Coulter, United States) flow cytometer using corresponding channels in three independent experiments. Values of the median were used for calculation. Results were represented as the mean ± SEM of three experiments.

### Measurement of LysoTracker Intensity

A day after seeding, cells were treated by ecdysterone (0, 250, 500 or 750 µM) for 48 h in triplicates. Before analysis, cells were treated with 75 nM LysoTracker Red DND-99 (ThermoFischer, United States) for 2 h at 37°С in a CO_2_ incubator. Then cells were washed in PBS, detached with trypsin and analyzed by flow cytometry (CytoFlex, Beckman Coulter, United States). Values of the median were used for calculation. Results were represented as the mean ± SEM of three experiments.

### Analysis of Autophagic Flux

Autophagic flux was revealed by blocking autophagy using chloroquine followed by western-blot with anti-LC3 and p62 antibodies, as well as immunofluorescence (staining with anti-LC3 antibodies). The next day after being planted in Petri dishes or glass cover slips, cells were treated with 0–1,500 µM Ecdy for 32 h followed by a co-treatment with the same concentrations of Ecdy and 50 µM chloroquine to block autophagy for 16 h. Then cells were subjected to either western-blot or immunofluorescence.

### Western-Blot

Cell lysates were prepared using RIPA buffer (150 mM NaCl; 50 mM Tris–HCl pH 8.0; 0.5%NP-40; 1 mM PMSF, protease inhibitor cocktail). After protein quantification by BCA assay (ThermoFischer, United States), 20 ug of Laemli-diluted cell lysates were loaded on 17% SDS-PAGE, run on TRIS-Glycine running buffer, followed by transfer to a nitrocellulose membrane (Bio-RAD, United States). Following 1 h blocking in PBST-diluted 5% nonfat milk, membranes were incubated with primary antibodies: LC3B (1:1,000, #2775S, Сell Signaling, United States), p62 (1:1,000, #5114, Сell Signaling, United States) or β-actin (1:5000, A3854, Sigma-Aldrich, United States). After PBST washing, secondary anti-mouse or anti-rabbit antibodies (1:10,000; Sigma-Aldrich, United States) conjugated with horseradish peroxidase were used. ECL system (ThermoScientific, United States) and ChemiDoc Touch Imager (Bio-Rad, United States) were applied for detection.

### Immunofluorescence

Cells grown on glass cover slips were fixed with 4% PFA in PBS for 15 min and then washed three times in PBS, followed by 60 min incubation in permeabilization blocking solution (5% BSA, 0.3% Triton X-100 in PBS) at room temperature. Then, cells were stained with anti-LC3B antibodies (1:200, #2775S, Сell Signaling, United States) in permeabilization blocking solution for 16 h at 4°C, washed three times in PBS, incubated with the secondary antibody in permeabilization blocking solution (goat anti-rabbit, AlexaFluor488, Invitrogen, United States) for 1 h at room temperature and washed three times in PBS. Slides were mounted using ProLong Gold Antifate Mountant with DAPI (P36931, Invitrogen). Images were analyzed by confocal microscope (Olympus, FV3000, Germany).

### Assessment of Drug Synergy

IC50 and drug synergy were obtained using results of MTT-assay. IC50 was calculated using AAT Bioquest IC50 online calculator https://www.aatbio.com/tools/ic50-calculator. Drug interaction was assessed by Chou-Talalay algorithms (CompuSyn software, http://www.combosyn.com/; (Chou and Talalay, 1984; Сhou, 2006). Results were represented as CI (Combination Index) plots and a Table which includes values for CI and DRI (Dose Reduction Index). CI < 1 attests synergistic action of drugs; DRI estimates the extent to which the dose of one or more agents in the combination can be reduced to achieve effect levels that are comparable with those achieved with single agents.

### Statistical Analysis

All results are demonstrated as mean ± standard deviation (SD) or standard error of the mean (SEM) of at least three biological replicates. Statistical significance was analyzed using the Student’s *t*-test: *p < 0.05; **p < 0.01; n.s – not significant.

## Results

### Ecdysterone inhibits Proliferation and Induces Death of Breast Cancer Cell Lines

Several papers describe the ecdysterone-mediated tumor suppressive effect on some cancer cells (Konovalova et al., 2002; Martins et al., 2015). We evaluated the effect of this drug on three human breast cancer cell lines with different molecular properties: MCF7 (luminal, ER^+^Pr^+^Her2^−^), MDA-MB-231 (TNBC) and MDA-MB-468 (TNBC).

**FIGURE 1:**
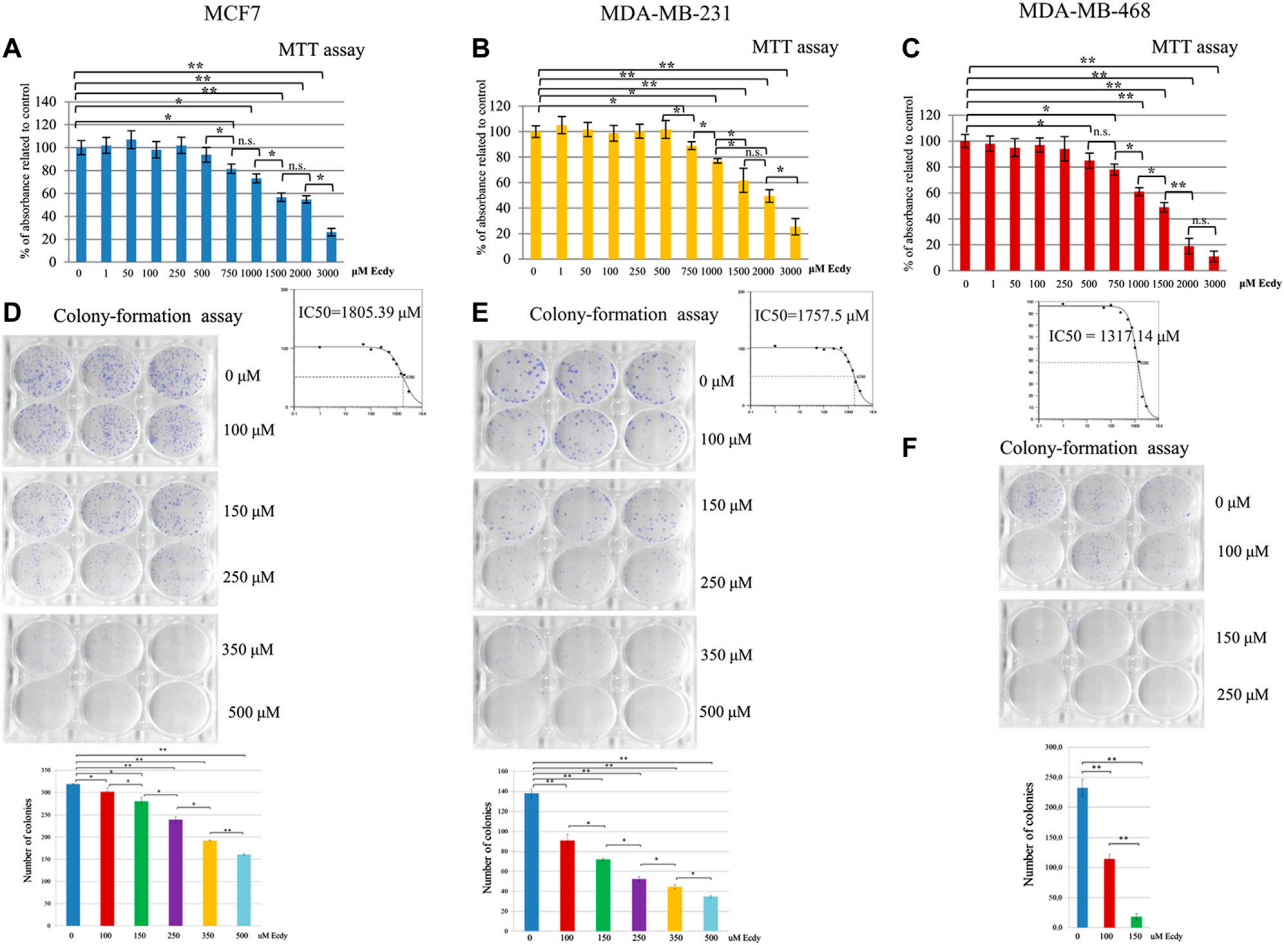
Ecdysterone inhibits breast cancer cell lines. Results of MTT-test for: **(A)** MCF7, **(B)** MDA-MB-231, and **(C)** MDA-MB-468 cells treated with increasing concentrations (0–3,000 µM) of Ecdy for 48 h. Results are depicted as the mean ± SD. *p < 0.05; **p < 0.01; n.s. – not significant. Corresponding IC50 plots are displayed below the MTT diagrams. IC50 was calculated using AAT Bioquest IC50 online calculator https://www.aatbio.com/tools/ic50-calculator. Results of the colony-formation assay for **(D)** MCF7, **(E)** MDA-MB-231, and **(F)** MDA-MB-468 treated with indicated concentration of Ecdy. Photographs of plates and quantification diagrams are shown. Results are displayed as the mean ± SEM of three experiments. *p < 0.05; **p < 0.01.

As ecdysterone is reported to exert anabolic activity in muscle tissue, which should facilitate the proliferation, we decided to carry out MTT assay to see if ecdysterone affects the proliferation of cancer cell lines studied. To do this, we have used a broad range of concentrations ranging from one to 3,000 µM. Results shown in Figures 1A–C clearly demonstrate that in our case the treatment with Ecdy has down-regulated all three cell lines starting with a concentration of 250–750 µM. No increase in cell proliferation was detected.

We have also carried out colony-formation assay. As this analysis implies the growth of colonies from single cells and they are very sensitive to any treatment, we have chosen a lower concentration of Ecdy. Results shown in Figures 1D–F confirm that the treatment with Ecdy inhibits the growth of all three cell lines. Moreover, photographs of plates with colonies clearly showed that not only the number of colonies, but also their size, was significantly reduced upon treatment with Ecdy.

**FIGURE 2:**
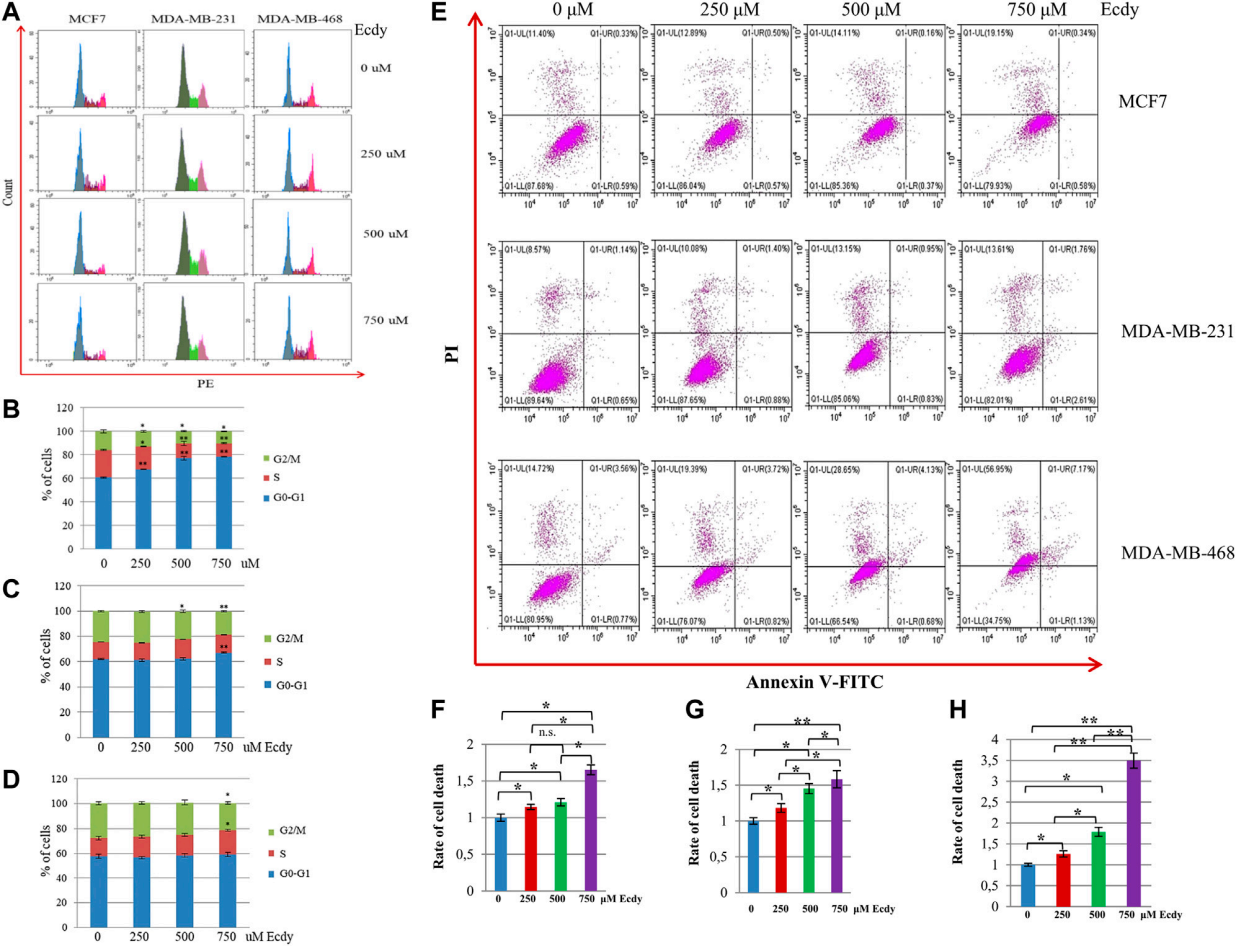
Ecdysterone alters the cell cycle and induces cell death depending on the cancer cell line. **(A)** Cell cycle profiles (flow cytometry) of breast cancer cell lines treated with 0, 250, 500 or 750 µM Ecdy for 48 h. Cell cycle analyses are shown for MCF7 **(B)**, MDA-MB-231 **(C)**, and MDA-MB-468 **(D)** cells. Results are displayed as the mean ± SEM of three experiments. *p < 0.05; **p < 0.01. **(E)** Annexin V-FITC/Propidium iodide (PI) profile of the same breast cancer cell lines treated with 0, 250, 500 or 750 µM Ecdy for 48 h (flow cytometry). Rates of cell death related to DMSO-treated cells (0 µM Ecdy) calculated for **(F)** MCF7, **(G)** MDA-MB-231, and **(H)** MDA-MB-468 cells are represented as diagrams. Results are calculated as mean ± SEM of three experiments. *p < 0.05; **p < 0.01; n.s – not significant.

The analysis of the cell cycle has shown that ecdysterone affected to varying degrees the cell cycle distributions of MCF7 and MDA-MB-231 cells, but had no effect on MDA-MB-468 cells (Figures 2A–D). It significantly increased the number of MCF7 cells in the G1-phase (Figure 2B). To a lesser extent, the same was true for MDA-MB-231 cells (Figure 2C). However, MDA-MB-468 cells were insensitive to Ecdy-induced alterations in the cell cycle (Figure 2D).

We also determined if ecdysterone affected the level of cell death. Annexin V/PI staining followed by flow cytometry (Figure 2E) demonstrated that ecdysterone increased the rate of cell death up to 1.6 fold for both MCF7 and MDA-MB-231 cells (Figures 2F,G), and 3.5 times in the case of MDA-MB-468 cells (Figure 2H). It is important to notice that although Ecdy elevated overall cell death in all cell lines, it did not increase the population of Annexin V-positive cells, suggesting that Ecdy elicited death of MCF7 cells via mechanism(s) other than apoptosis (Figure 2E). In contrast, Ecdy increased the population of Annexin V-positive (apoptotic) cells by 30% for MDA-MB-231 and by 48% for MDA-MB-468 cell line (Figure 2E).

These data suggest that the ability of ecdysterone to induce cell cycle arrest or elicit cell death of breast cancer cells presumably depends on the genetic background of a particular cell line.

### Ecdysterone Down-Regulates Energy Metabolism of Breast Cancer Cells

As ecdysterone possesses anabolic properties in muscle tissue, we were interested to see whether it alters the energy metabolism of the breast cancer cell lines. To do this, we employed the SeaHorse energy profiling using Energy Phenotype kit.

**FIGURE 3:**
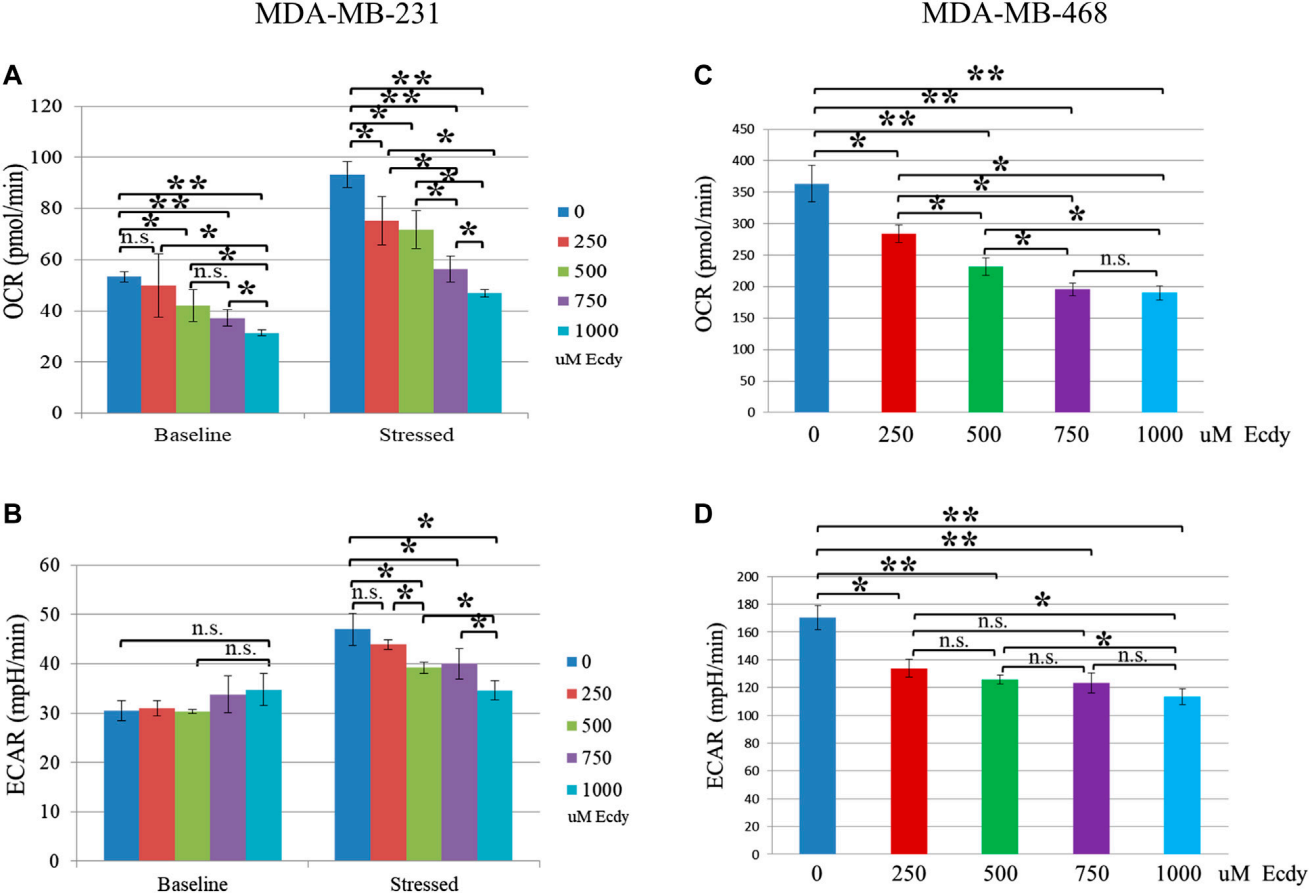
Ecdysterone attenuates energy metabolism of TNBC breast cancer cell lines. SeaHorse energy profiling (Energy Phonotype kit) of **(A,B)** MDA-MB-231 and **(C,D)** MDA-MB-468 cancer cell lines treated with 0, 250, 500, 750 or 1,000 µM Ecdy for 48 h. OCR–oxygen consumption rate (respiration), ECAR–extracellular acidification rate (glycolysis). Baseline–base (normal) respiration (OCR) or glycolysis (ECAR); Stressed - OCR and ECAR of cells under an induced energy demand (in the presence of stressor compounds–FCCP and olygomycin). Stressed OCR and ECAR reflect metabolic potential–percentage increase of stressed OCR over baseline OCR, and stressed ECAR over baseline ECAR. Metabolic Potential is the measure of cells’ ability to meet an energy demand via respiration and glycolysis. Results are shown as mean ± SEM of three experiments. *p < 0.05; **p < 0.01; n.s – not significant.

Energy profiling of MDA-MB-231 cells treated with 0, 250, 500, 750 or 1,000 μM of ecdysterone has shown ecdysterone-mediated inhibition of respiration (basic OCR). For instance, 500 and 750 μM Ecdy decreased respiration by approximately 21 and 31%, respectively (Figure 3A). At the same time, ecdysterone did not alter glycolysis (basic ECAR, Figure 3B) but significantly decreased the metabolic potential (both stressed respiration (stressed OCR) and glycolysis (stressed ECAR), Figures 3A,B). Thus, 500 μM Ecdy mitigated stressed OCR by 23% and stressed ECAR by 18%. Stressed OCR and ECAR reflect the metabolic potential of cells–percentage increase of stressed OCR over the baseline OCR, and stressed ECAR over the baseline ECAR. Metabolic Potential (MP) is the measure of cells’ ability to meet the energy demand via respiration and glycolysis. Thereby, these results suggest that ecdysterone greatly reduced the capacity of MDA-MB-231 cells for metabolic adaptation.

In the case of MDA-MB-468 cell line, the same treatment with ecdysterone has led to an inhibition of both the baseline respiration and of glycolysis (Figures 3C,D). Even 250 μM Ecdy dampened respiration and glycolysis by 22%, whereas 750 μM Ecdy inhibited them further by 47 and 28%, respectively. Taken together, these data suggest Ecdy was able to attenuate the level of energy metabolism in TNBC breast cancer cells.

### Ecdysterone Induces Autophagy

**FIGURE 4:**
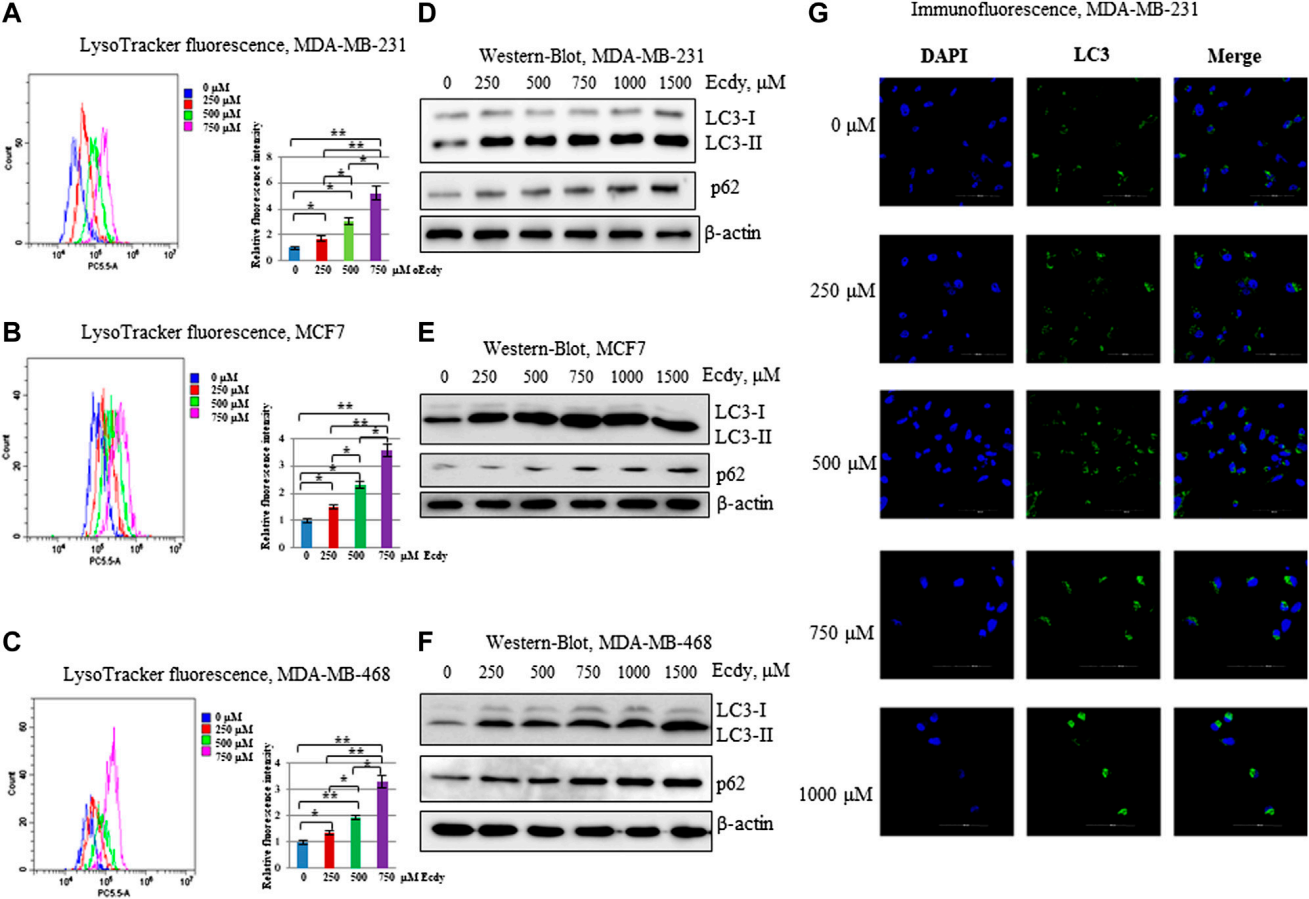
Ecdysterone induces autophagy. **(A)** MDA-MB-231, **(B)** MCF7, **(C)** MDA-MB-468 cells were treated with 0, 250, 500, 750 µM Ecdy for 48 h followed by LysoTracker staining and flow cytometry analysis. The fluorescence of LysoTracker reflects the amount of lysosomes. Flow cytometry plots with median fluorescence and diagrams with fluorescence intensity related to DMSO-treated cells (0 µM Ecdy) are depicted. Results are calculated as the mean ± SEM of three experiments. *p < 0.05; **p < 0.01. **(D)** MDA-MB-231, **(E)** MCF7, **(F)** MDA-MB-468 cell lines were treated with 0, 250, 500, 750, 1,000, and 1,500 µM Ecdy for 32 h and then co-treated with the same concentrations of Ecdy and 50 µM chloroquine to block autophagy for 16 h followed by western-blot analysis for LC3 and p62 reflecting autophagy flux. **(G)** MDA-MB-231 cells treated as above were stained with DAPI and anti-LC3 antibodies and analyzed by immunofluorescence microscopy.

Tang and colleagues (Tang et al., 2018) has reported that ecdysterone promotes autophagy in osteoporotic rats. We have carried out staining in three ecdysterone-treated (0, 250, 500, and 750 μM, 48 h) breast cancer cell lines with LysoTracker, a fluorescent dye, reflecting the number of lysosomes which can be indicative of autophagy (Chikte et al., 2014). Flow cytometry analysis has shown the strong increase of LysoTracker fluorescence in all three cell lines in order of increase the ecdysterone concentration (Figures 4A–C).

To further study whether ecdysterone affects the autophagic flux the breast cancer cell lines were incubated with different concentrations of ecdysterone (0, 250, 500, and 750 μM) for 32 h followed by 16 h of incubation with both ecdysterone and 50 μM chloroquine to block the autophagic-mediated degradation. Treated cells were analyzed by western-blot with LC3 and p62 antibodies or by immunofluorescence microscopy to evaluate the LC3 staining. Results of both immunoblotting and immunofluorescence (Figures 4D–G; Supplementary Figure S1) demonstrate that in the case of all three lines, ecdysterone strongly induced autophagy.

Taken together, these data strongly suggest that ecdysterone induces autophagy in all breast cancer cells concomitantly with the increasing ecdysterone concentration.

### Ecdysterone synergizes With Doxorubicin to Down-Regulate Breast Cancer Cell Lines

Given that ecdysterone mediated the inhibition of growth of all breast cancer cell lines, we decided to examine its effect on cancer cells a part of the combined treatment with doxorubicin, a genotoxic drug which is widely applied as chemotherapeutics. To this end, we carried out MTT-assay using ecdysterone (250, 500, or 750 μM) and doxorubicin (0.15 or 0.25 μM) alone or in combination. To determine its synergistic effect we calculate the combination Index (CI) and dose reduction index (DRI) using Chou-Talalay algorithms (Chou and Talalay, 1984; Chou, 2006).

**FIGURE 5:**
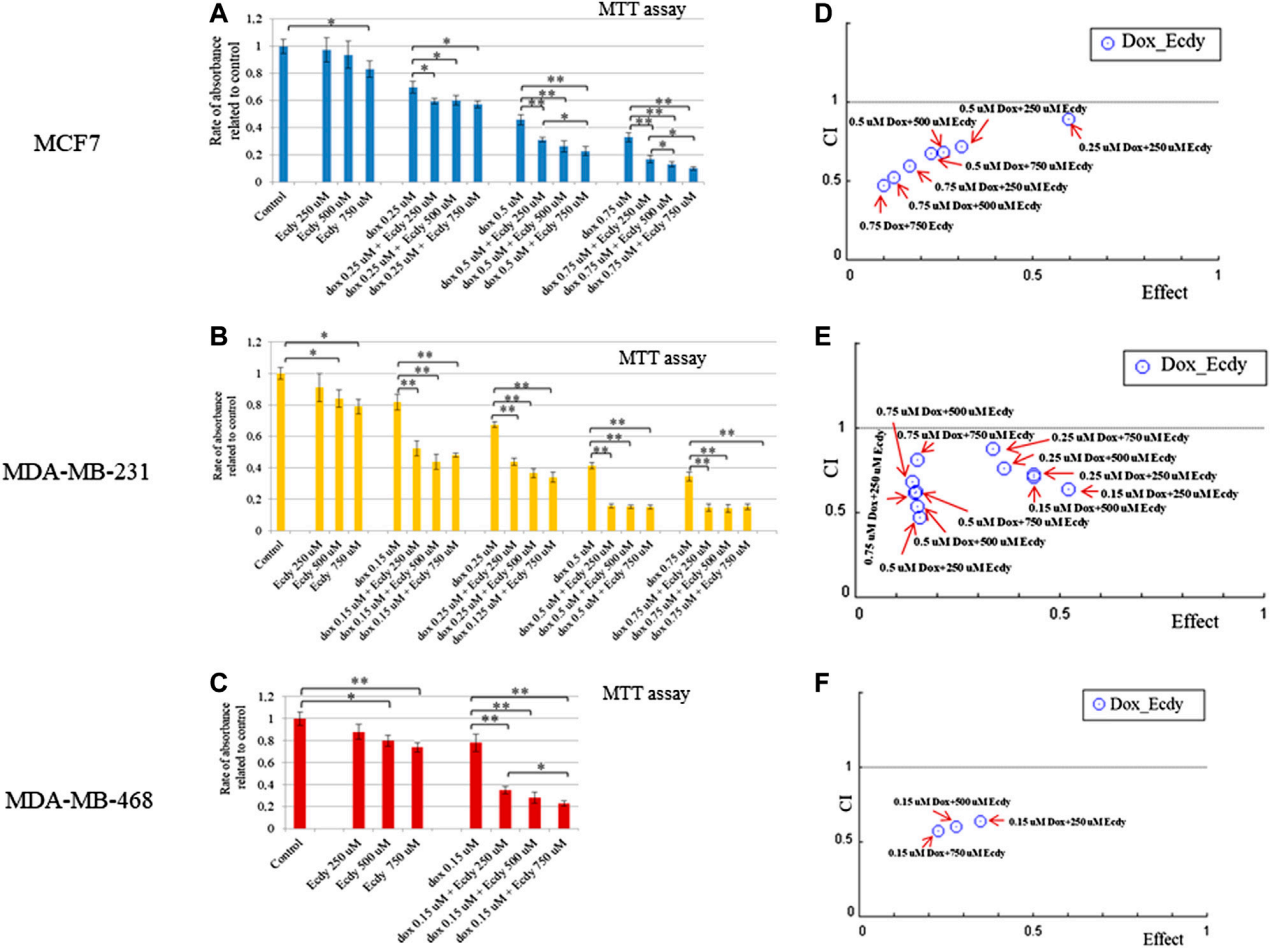
Ecdysterone acts synergistically with doxorubicin to inhibit breast cancer cells. Results of MTT-test demonstrating the inhibition of **(A)** MCF7, **(B)** MDA-MB-231 **(C)** MDA-MB-468 cell lines by doxorubicin or ecdy along and in combination. Results are represented as the mean ± SD. *p < 0.05; **p < 0.01. Combination Index (CI) plots which reflect the distribution of CIs for different combinations of Dox/Ecdy concentrations, depending on their effects (rate of absorbance related to control) for: **(D)** MCF7, **(E)** MDA-MB-231 **(F)** MDA-MB-468 cells. CI plots were calculated using Chou-Talalay algorithms (CompuSyn software, http://www.combosyn.com/; Сhou, 2006). CI < 1 attests at the synergistic action of drugs; DRI estimates the extent to which the dose of doxorubicin in the combination with the indicated dose of Ecdy can be reduced to achieve effect levels that are comparable to those achieved with single agents.

The results shown in Figures 5A–C demonstrate that ecdysterone significantly sensitizes all breast cancer cell lines to the treatment with doxorubicin. CI plots (Figures 5D–F) and Table 1 show that all three Ecdy concentrations have a pronounced synergistic (CI ranges 0.47–0.89) interaction with doxorubicin. In turn, the use of Ecdy allowed the reduction of the effective dose of doxorubicine from 1.4 to 17.9 times (Table 1), depending on the particular cell line. It is important to notice that in most cases the addition of even 250 μM ecdysterone was sufficient to down-regulate the growth of breast cancer cells 1.5–2 times more efficiently than the corresponding concentrations of doxorubicin (Figures 5A–C).

**TABLE 1:** Synergistic effect of ecdysterone and doxorubicin calculated using Chou-Talalay algorithms (Chou and Talalay, 1984; Chou, 2006).

	Dose dox, µM	Dose ecdy, µM	Effect	CI	Dox_DRI
MCF7	0,25	250	0,59	0,89	1,36
	0,25	500	0,6	1,07	3,04
	0,25	750	0,57	1,13	2,17
	0,5	250	0,31	0,72	11,66
	0,5	500	0,26	0,68	6,63
	0,5	750	0,23	0,67	4,88
	0,75	250	0,17	0,59	17,86
	0,75	500	0,13	0,52	10,58
	0,75	750	0,1	0,47	8,26

	Dose dox, µM	Dose ecdy, µM	Effect	CI	Dox_DRI
MDA-MB-231	0,15	250	0,52	0,64	2,61
	0,15	500	0,44	0,72	3,32
	0,15	750	0,48	1,04	2,93
	0,25	250	0,44	0,72	1,98
	0,25	500	0,37	0,77	2,45
	0,25	750	0,34	0,88	2,65
	0,5	250	0,16	0,47	2,64
	0,5	500	0,15	0,54	2,76
	0,5	750	0,15	0,62	2,79
	0,75	250	0,14	0,61	1,89
	0,75	500	0,14	0,68	1,95
	0,75	750	0,15	0,81	1,84

	Dose dox, µM	Dose ecdy, µM	Effect	CI	Dox_DRI
MDA-MB-468	0,15	250	0,35	0,64	1,69
	0,15	500	0,28	0,6	1,85
	0,15	750	0,23	0,57	1,99

CI, Combination Index, Dox_DRI, Dose Reduction Index for doxorubicin.

These data clearly demonstrate the ability of ecdysterone to synergize with doxorubicin to down-regulate the proliferation of breast cancer cells.

### Ecdysterone Significantly Enhances Doxorubicin- and 2-DG-Induced Cell Death

The observed synergistic effect of doxorubicin and ecdysterone likely results from cell death. To directly check this hypothesis, we treated the cell lines with ecdysterone (250 μM) and doxorubicin (0.15 or 0.25 μM) alone or in combination, followed by staining with Annexin V/7AAD and flow cytometry analysis. Since we have already shown that ecdysterone down-regulated the metabolic potential, we decided to apply 2-deoxyglucose (2-DG), a promising inhibitor of glycolysis, which currently undergoes clinical trials. We also treated cells with either 2-DG (10 mM) or ecdysterone (250 μM) alone or in combination.

**FIGURE 6:**
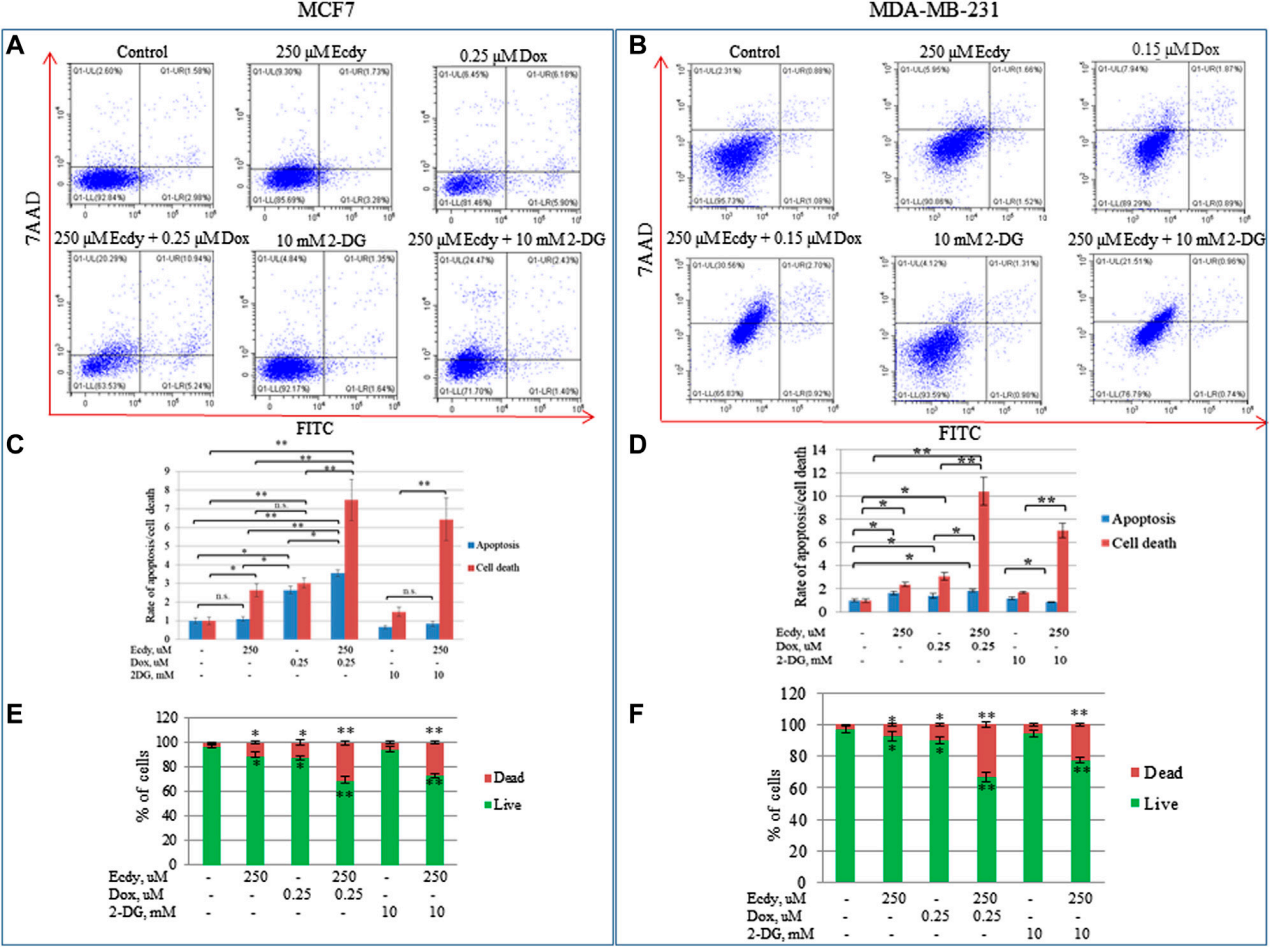
Ecdysterone synergizes with doxorubicin and 2-DG to induce apoptosis and death of breast cancer cell lines. Annexin V-FITC/7-AAD profiles of **(A)** MCF7, **(B)** MDA-MB-231 breast cancer cells treated with doxorubicin, ecdysterone, and 2-DG alone or in combination for 48 h (flow cytometry). **(C,D)** Diagrams show the rate of apoptosis/total cell death for the same cell lines based on Annexin V-FITC/7-AAD data. **(E,F)** Diagrams show percent of live/dead cells. Results are calculated as the mean ± SEM of three experiments. *p < 0.05; **p < 0.01; n.s – not significant.

Figures 6A–F and Supplementary Figures S2A–C demonstrate that the combined treatment (either doxorubicin or 2-DG with 250 μM Ecdy) both increased the level of cell death by several times relative to control, or in comparison to the treatment with an individual drug. Accordingly, co-treatment with doxorubicin and ecdysterone elevated the level of apoptosis in MCF7 cells by 23% and in MDA-MB-231 cells by 3.15 times, respectively, compared to doxorubicin alone.

Co-treatment of Ecdy with 2-DG also significantly enhanced both apoptosis and total cell death (Figures 6C–F). It increased the rate of cell death by 21% for MCF7 and 17% for MDA-MB-231 cells.

**FIGURE 7:**
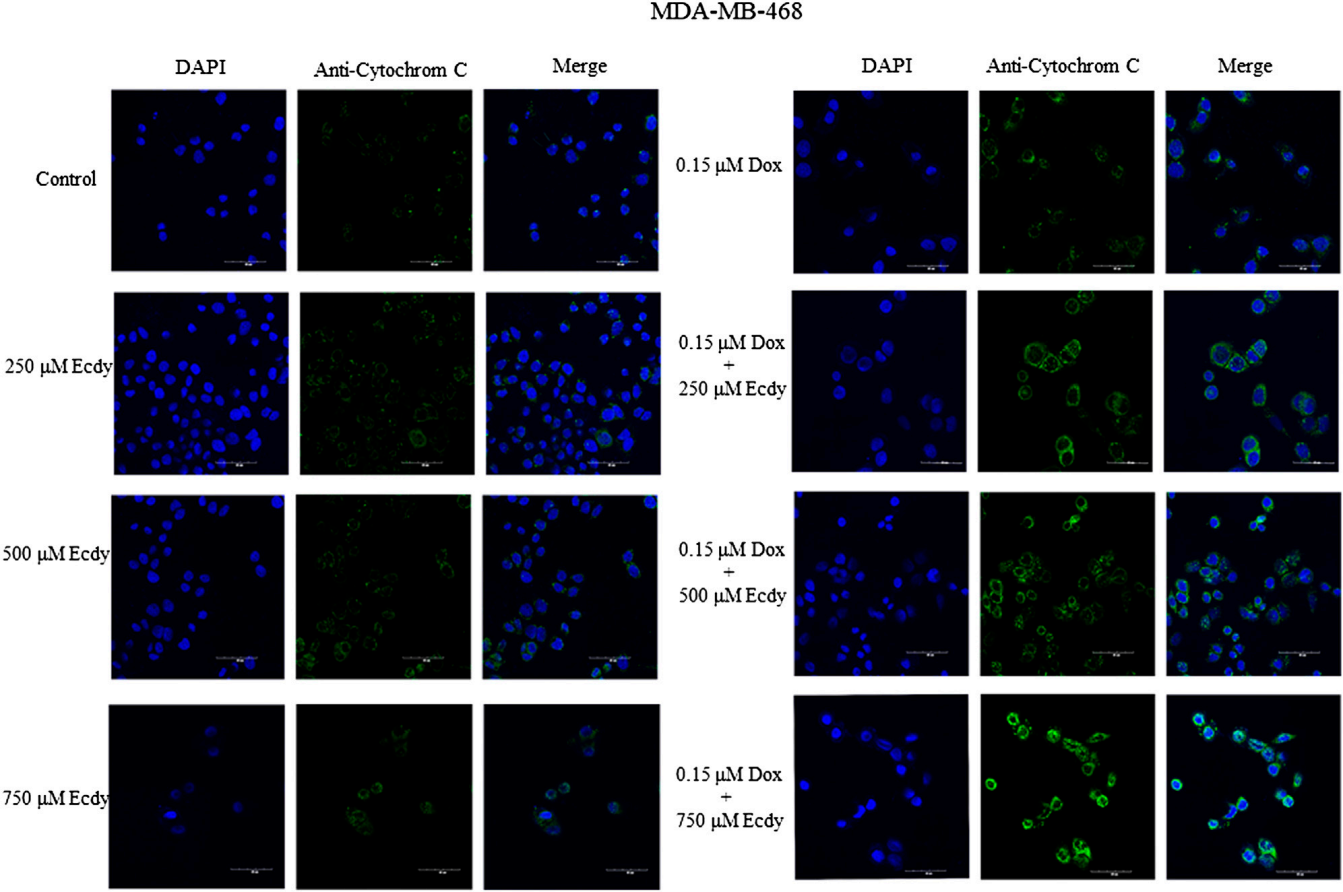
Ecdysterone synergizes with doxorubicin to induce apoptosis in MDA-MB-468 cells. Confocal microscopy showing an increase in cytochrome C release upon combined treatment with doxorubicin and Ecdy. MDA-MB-468 cells treated with 0.15 µM doxorubicin or 250–750 µM Ecdy for 48 h alone or in combination were stained with DAPI and anti-cytochrome C antibodies and subsequently analyzed by confocal microscopy.

We also repeated the previously described treatment of several breast cancer cells with combinations of doxorubicin (0.25 or 0.15 μM, respectively) and ecdysterone (250, 500, and 750 μM) followed by immunofluorescence microscopy to detect the release of cytochrome C from the mitochondria upon apoptosis. Taken together, results shown in Figure 7 and Supplementary Figures S2, S3 confirm that treatment with Ecdy significantly enhanced the doxorubicin-induced release of cytochrome C in all cancer cell lines.

Taken together, these data suggest that ecdysterone mediates strong synergy with doxorubicin in attenuation of the proliferation of breast cancer cells.

### Ecdysterone Has Minimal Effects on Non-Transformed Human Fibroblasts

To compare the effects of ecdysterone on cancerous vs. non-cancerous cells, we decided to test normal non-transformed human fibroblasts, DF2 and WI-38 cell lines after the treatment with Ecdy.

First, we assessed the effect of Ecdy on proliferation of breast cancer cell lines and non-transformed human fibroblasts. As shown in Supplementary Figure S4A,B, Ecdy down-regulated the proliferation of cancer cell lines significantly more robustly compared to normal human fibroblasts at all concentrations tested.

To study whether ecdysterone elicits effects on autophagy in non-transformed fibroblasts similar to what we have observed in breast cancer cells, we employed the previously described flow cytometry analysis with LysoTracker DND-99 Red combined with western blotting for LC3 and p62. Results shown in Supplementary Figure S6 demonstrate that Ecdy induced autophagy in fibroblasts to a much lesser extent than in breast cancer cells (Figure 4).

We have also investigated the effect of combined treatment with Ecdy and doxorubicin on DF2 and WI38 fibroblasts (Supplementary Figure S7). To this end, we have employed previously described Annexin V-FITC/7-AAD staining followed by flow cytometry. Surprisingly, the minimal effective concentration of Ecdy (250 uM) in combination with doxorubicin that induced death of breast cancer cells, had almost no effect on DF2 and WI38 fibroblasts (Figure 6; Supplementary Figure S2).

Finally, we have carried out MTT assay on DF2 and WI38 normal human fibroblasts as well as mouse embryonic fibroblasts (MEFs) treated with either Ecdy alone or in combination with doxorubicin. Supplementary Figure S8 demonstrates that in contrast to breast cancer cell lines (Figure 5), an increased ecdysterone concentration up-regulated the survival of WI-38 and MEFs cells (Supplementary Figures S7B,C). In the case of DF2 cells, the combined treatment (doxorubicin + ecdysterone) displayed even a small protection of these cells from doxorubicin (Supplementary Figure S7A). Regarding WI-38 and MEFs (Figures 7B,C), the same combined treatment has incomparable low inhibitory effect in contrast to breast cancer cells (Figure 5).

Taken together, our experiments show that ecdysterone significantly down-regulates cancer cells with no or little effect on fibroblasts.

## Discussion

Ecdysterone is a hormone found in arthropods, yet is also synthesized by a number of plants to combat insect pests by disrupting their development, molting, and reproduction. Unlike insects, mammals do not harbor any homologs of ecdysterone nuclear receptor (EcR). However, ecdysterone possesses a variety of beneficial pharmacological effects on humans, including anabolic and adaptogenic ones (Báthori et al., 2008). Ecdysterone is marketed as a diet supplement to enhance the physical performance of athletes, and recently became the focus of WADA investigations (https://www.wada-ama.org/en/resources/research/ecdysteroids-as-non-conventional-anabolic-agents-pharmacodynamics; Parr et al., 2020). Numerous studies have documented oncogenic properties of male steroid hormones and its derivatives on several human organs, including testis, liver, breast, and others (Sirianni et al., 2012; Salerno et al., 2018). Therefore, it is important to assess all biologically active supplements for their potential side effects including the tumorigenic one.

Although positive ecdysterone-mediated pharmacological influence on organisms is well documented (Lafont and Dinan, 2003), we decided to examine possible pharmacological effects of ecdysterone on proliferation of human breast cancer cell lines of different molecular subtypes. Surprisingly, despite the fact that anabolic properties of ecdysterone in muscles have been reported (Parr et al., 2015; Isenmann et al., 2019), we have not observed ecdysterone-mediated growth stimulation of cancer cells. Instead, in our MTT experiments administration of ecdysterone caused the attenuation of cell growth of breast cancer cells starting from the concentration of 250–750 μM. Apparently, Ecdy can negatively regulate cancer cells through various mechanisms because it inhibited the cell cycle and induced death to a different extend depending on the particular cellular background. While Ecdy significantly affected the cell cycle distribution of MCF7 cells, it had almost no effect on cell cycle of MDA-MB-468 cells. Furthermore, it elicited a two-fold increase in cell death of the MDA-MB-468 cells relative to MCF7. In contrast to cancer cells, ecdysterone displayed a significantly less inhibitory impact on proliferation of human non-transformed fibroblasts compared to cancer cells.

Metabolic reprogramming is a known hallmark of cancer cells, in which they manifest diverse metabolic phenotypes to maintain their proliferation and to combat anticancer therapies (Shuvalov et al., 2017; Sun et al., 2020). Among breast cancers, the TNBC subtype has the worst prognosis with questioned targeted therapies. Therefore, we decided to assess the influence of Ecdy on the energy metabolism of two TNBC cancer cell lines. The SeaHorse energy profiling has shown that Ecdy significantly dampened respiration, as well as the metabolic potential of MDA-MB-231 cells, and strongly reduced both respiration and glycolysis in MD-MB-468 cells. Moreover, we have revealed that Ecdy sensitizes breast cancer cell lines to 2-DG which is in accordance with Ecdy-mediated down-regulation of energy metabolism. 2-DG is a promising inhibitor of glycolysis, which decreases the energy of the cancer cells thus making chemotherapy and other treatments more effective. It underwent clinical trials and most likely is useful for the treatment of breast cancer including TNBC (Wokoun et al., 2017; Lucantoni et al., 2018; O’Neill et al., 2019).

Although Ecdy only weakly inhibits proliferation of fibroblasts in the proliferation assay, it does activate fibroblasts in MTT assay which may result from their metabolic activation. The effect of Ecdy on metabolism of different cancer and non-cancer cells should be studied in further details.

The modulation of cancer-specific metabolic adaptations weakens the malignant cells and widens the therapeutic window for effective treatment of TNBC patients (Lanning et al., 2017; Wang et al., 2020). Ecdy-mediated negative regulation of the energy metabolism in TNBC cells can be potentially important for the treatment of this most dangerous sub-type of breast malignancy.

Ecdy can promote autophagy upon the onset of osteoporosis in rats (Tang et al., 2018). In addition, Ecdy protects from degeneration human nucleus pulposus cells, which form the inner core of the vertebral disc (Wen et al., 2019). This effect is mediated by Ecdy-dependent induction of autophagy, which counteracts the effect of apoptosis. In line with these observations, we have demonstrated that Ecdy strongly induced autophagy in breast cancer cells, in contrast to non-transformed human fibroblasts. Although autophagy can play dual roles in both tumor promotion and suppression (Yun and Lee, 2018), in terms of chemotherapy autophagy is usually considered as a mechanism of drug-resistance against therapeutics. For example, doxorubicin-induced autophagy is involved in the development of chemoresistance, and the inhibition of autophagy effectively overcomes doxorubicin resistance in a variety of cancers (Zhou et al., 2019).

Surprisingly, despite its positive effect on autophagy, Ecdy displayed a strong synergistic effect (CI ranges 0, 47-0, 89) with doxorubicin, which significantly enhances doxorubicin-induced cell death (DRI ranges 1, 4-17, 9 times) of breast cancer cells according to Chou-Talalay algorithms (Chou and Talalay, 1984; Chou, 2006). Notably, Ecdy strongly enhanced the action of doxorubicin in concentrations (250, 500, and 750 µM), which are sufficient to inhibit energy metabolism and induce autophagy. It is important to note that when Ecdy was used alone, it failed to significantly down-regulate the proliferation of cancer cells. Noteworthy, Ecdy was not able to sensitize non-cancerous (fibroblast) cells to doxorubicin as it was observed for breast cancer cells. Our results are in accordance with other studies (Konovalova et al., 2002; Martins et al., 2012; Martins et al., 2015) that have shown that ecdysterone made both drug-resistant and non-drug-resistant cancer cells more susceptible to doxorubicin treatment. Furthermore, Ecdy was shown to significantly stimulate the chemotherapeutic effect of cisplatin in mice models (Konovalova et al., 2002). Taken together, these data suggests that in moderate concentrations, Ecdy sensitizes cancer cells to treatments with chemotherapeutic agents and thus can potentially serve as an adjuvant therapeutic.

Furthermore, since Ecdy enhances the ability to cope with stress and enhances resistance to tiredness (Báthori et al., 2008; Dinan et al., 2009), it seems beneficial to administer it as part of cytotoxic therapy with doxorubicin. The latter produces multiple severe side effects including the cumulative cardiotoxicity, acute nausea and vomiting, gastrointestinal disturbances, alopecia baldness, and neurologic disturbances (Carvalho et al., 2009). However, additional experiments aimed at the elucidation of effectiveness of Ecdy and its toxicity to organs and tissues are required to assess the therapeutic potential of ecdysterone as an adjuvant therapy to treat breast cancer.

## Data Availability Statement

All datasets presented in this study are included in the article/Supplementary Material.

## Author Contributions

NB and OS designed experiments and wrote the manuscript. OS carried out majority of experiments. OF and AD participated in MTT assays. AP had part in SeaHorse experiments. ET participated in IF studies. GJ contributed to western-blot experiments. All authors participated in preparation of the final version of the manuscript.

## Funding

OS, OF, ET, AP, AD, and YG acknowledge the support from RSF grant # 18-75-10076. OS, OF, AD, and NB appreciate the support of the grant from the Russian Government Program for the Recruitment of the leading scientists into the Russian Institutions of Higher Education 14. W03.31.0029. The authors also acknowledge the Ministry of Science and Higher Education of the Russian Federation (agreement # 075-00337-20-03, project FSMG-2020-0004). NB appreciates the support of RFBR, project #18-29-09144.

## Conflict of Interest

The authors declare that the research was conducted in the absence of any commercial or financial relationships that could be construed as a potential conflict of interest.

## Glossary

Ecdy: ecdysterone
Dox: doxorubicin
ERbeta: Estrogen Receptor beta
TNBC: Triple Negative Breast Cancer
CI: Combination Index
DRI: Dose Reduction Index
2-DG: 2-deoxyglucose
DMSO: dimethyl sulfoxide
FCCP: Carbonyl cyanide 4-(trifluoromethoxy)phenylhydrazone
PI: Propidium iodide
7-AAD: 7-Aminoactinomycin D
ECAR: Extracellular Acidification Rate
OCR: Oxygen Consumption Rate
MDR: Multiple Drug Resistance
