# Supplementary figures and images for: An Arthropod Hormone, Ecdysterone, Inhibits the Growth of Breast Cancer Cells via Different Mechanisms
Source: Front Pharmacol. 2020 Oct 30;11:561537. doi: 10.3389/fphar.2020.561537 (PMC7663021; doi:10.3389/fphar.2020.561537)

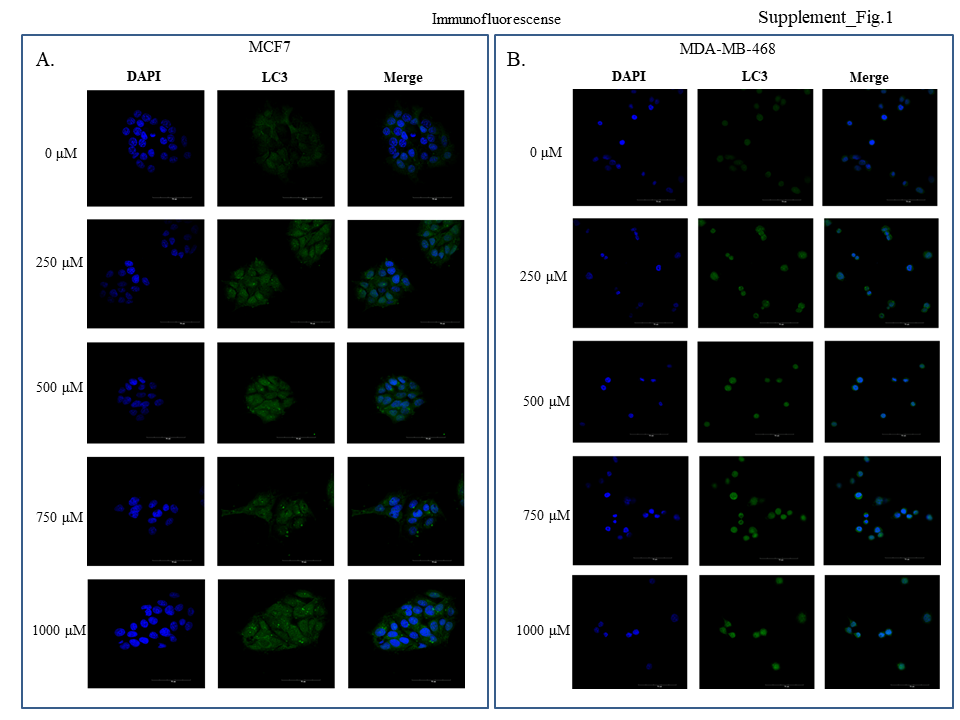

Supplement: Supplementary file 1 [file image1.tif]

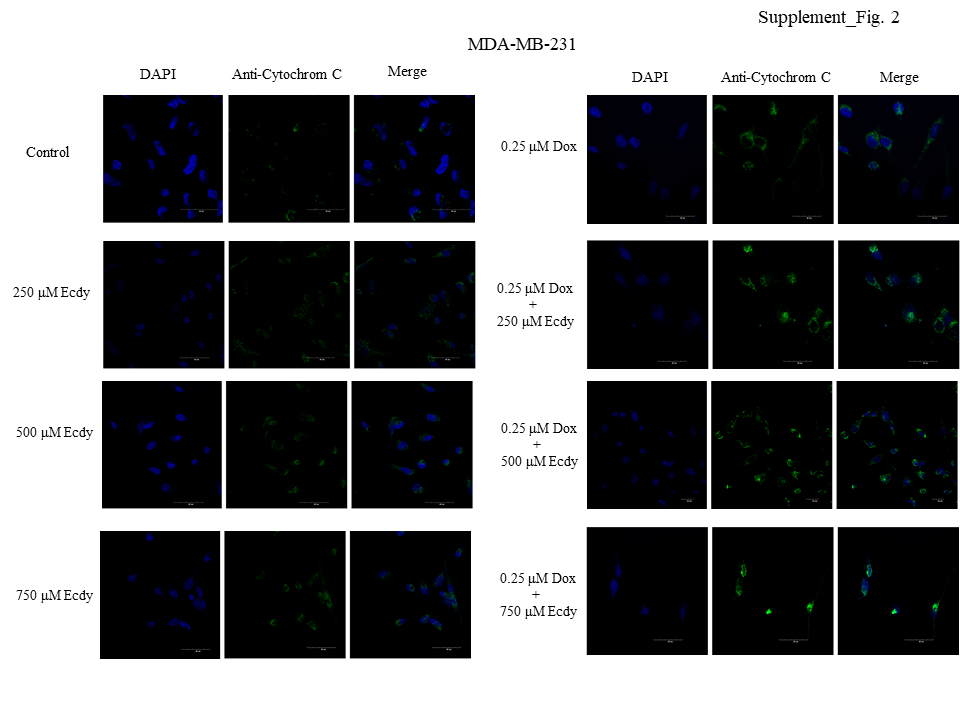

Supplement: Supplementary file 2 [file image2.tif]

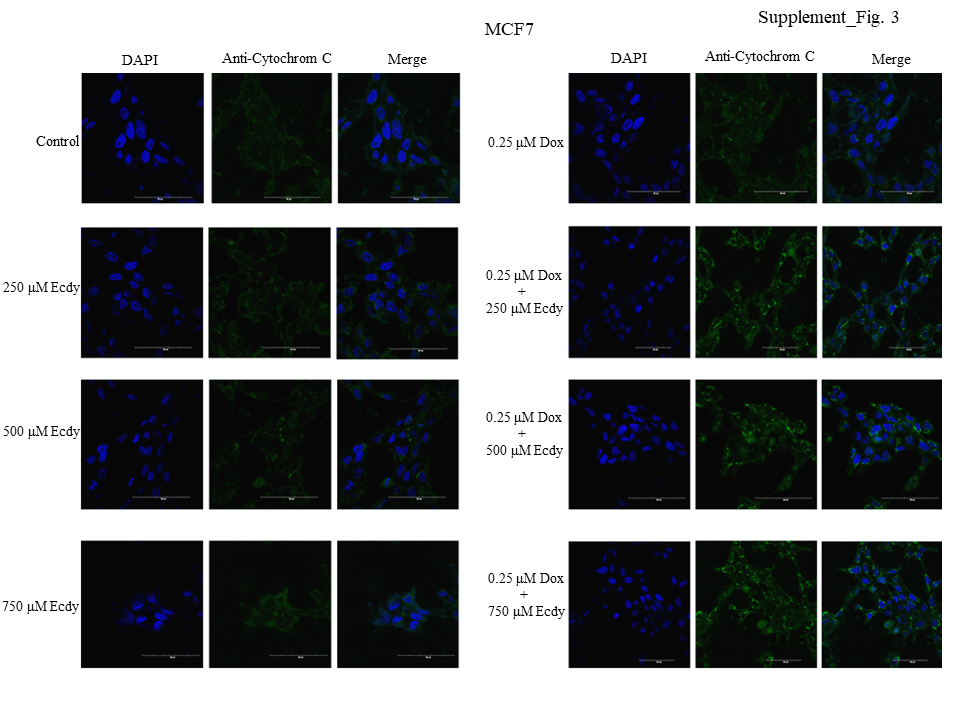

Supplement: Supplementary file 3 [file image3.tif]

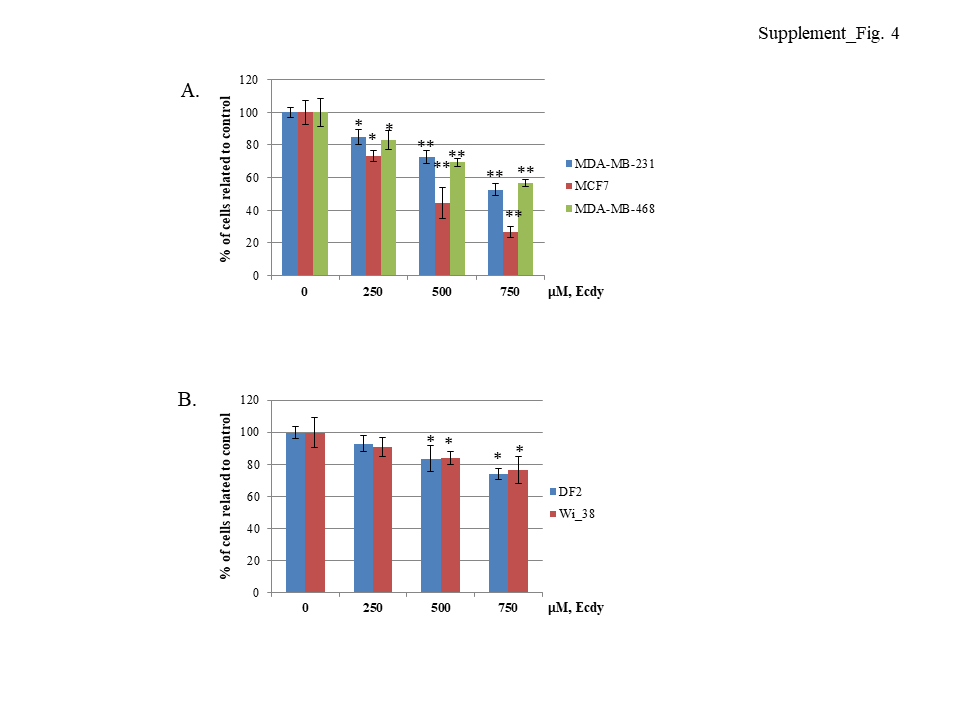

Supplement: Supplementary file 4 [file image4.tif]

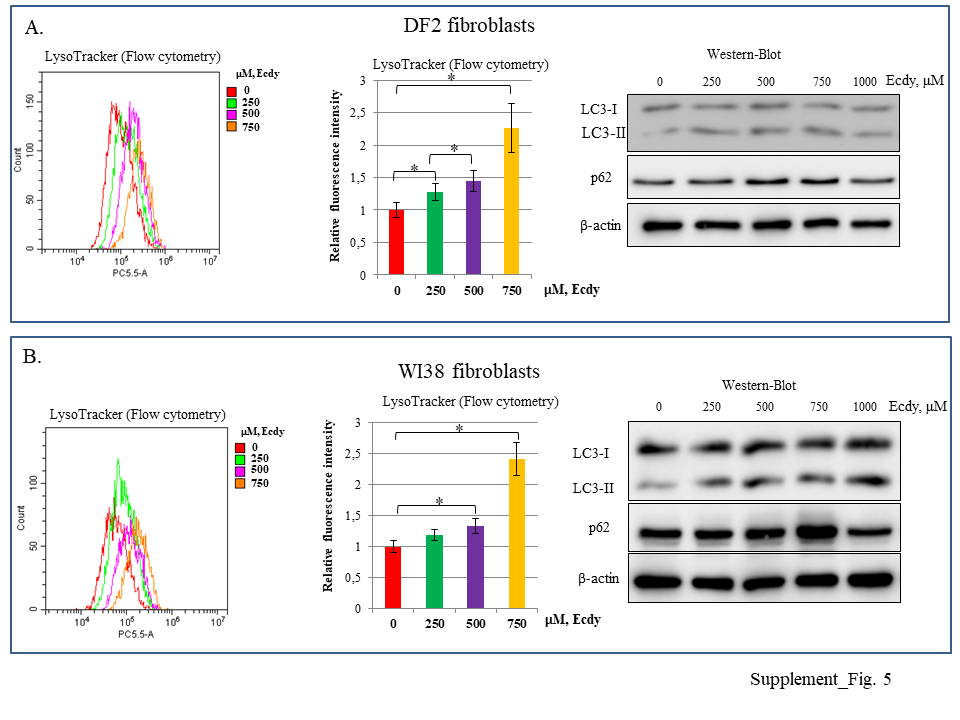

Supplement: Supplementary file 5 [file image5.tif]

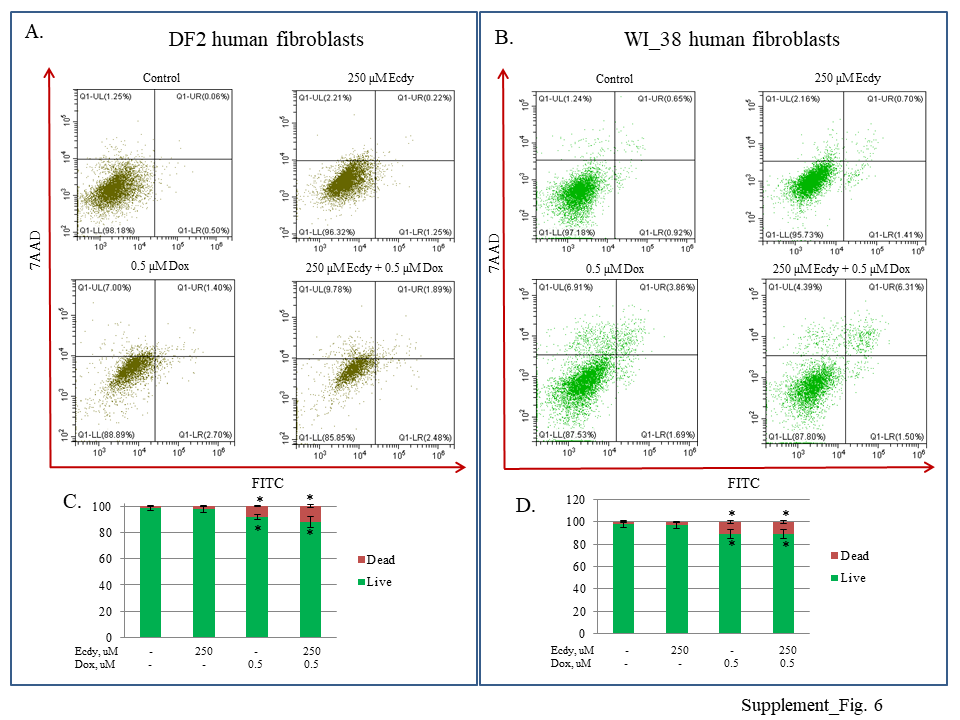

Supplement: Supplementary file 6 [file image6.tif]

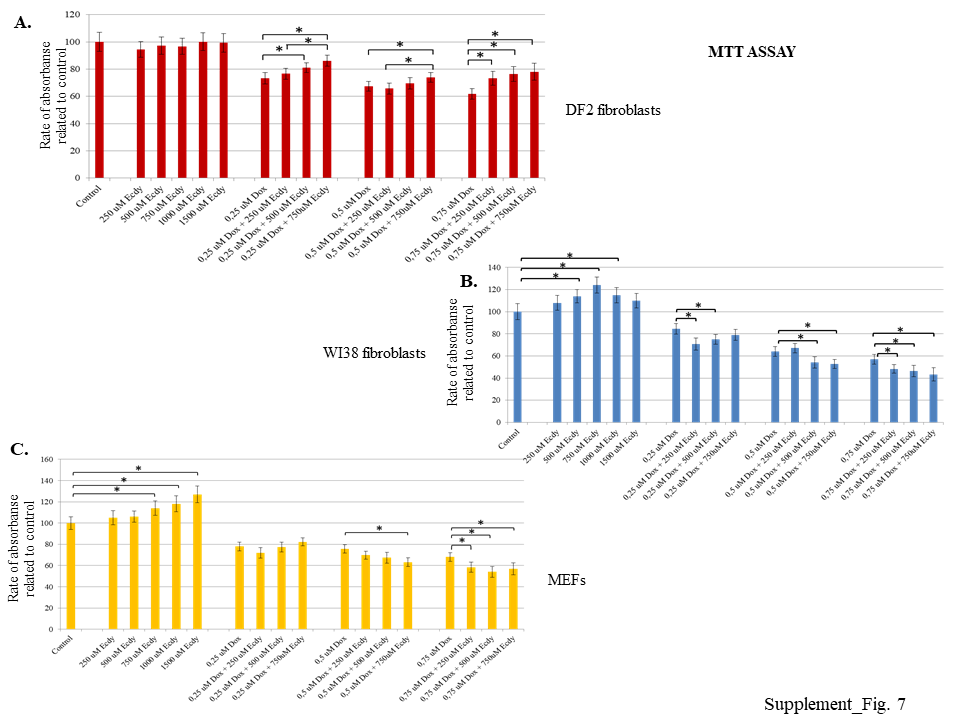

Supplement: Supplementary file 7 [file image7.tif]
